# Supplementary material for: A rapid antibody screening haemagglutination test for predicting immunity to SARS-CoV-2 variants of concern
Source: Commun Med (Lond). 2022 Apr 5;2:36. doi: 10.1038/s43856-022-00091-x (PMC9053181; doi:10.1038/s43856-022-00091-x)
Supplement: Supplementary file 1 — Supplementary Information [file 43856_2022_91_MOESM1_ESM.pdf]

Supplementary Figure 1. Correlations between haemagglutination test antibody endpoint titres and binding antibodies (anti-IgG spike and anti-IgG RBD)

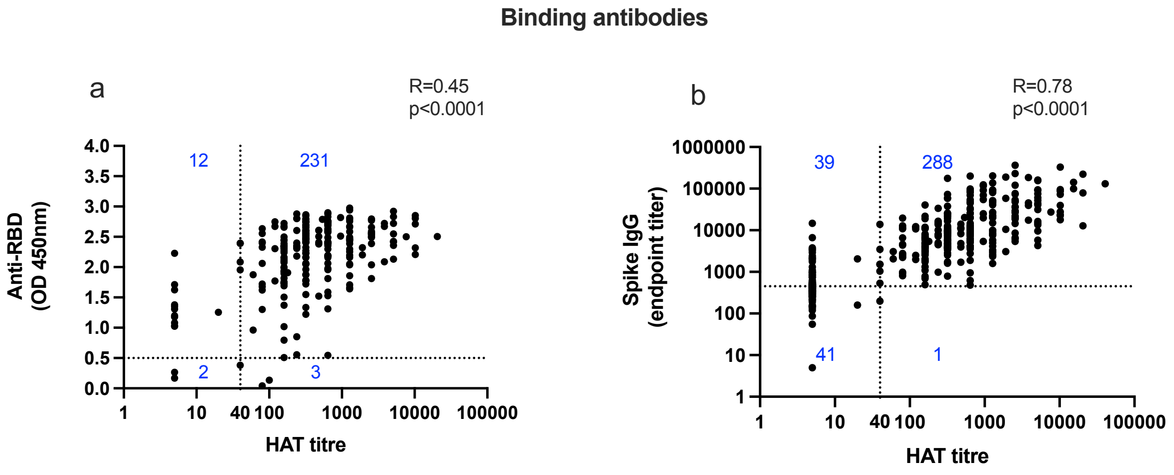

Correlation of endpoint HAT titres with binding antibody titres from a cohort of infected individuals from the first pandemic wave (diagnosis by PCR from nasopharyngeal swabs or serology in Bergen). Wuhan antibodies were measured by ELISA; **a** receptor binding domain (RBD) and **b** spike endpoint titres. The dotted lines show the lowest detectable titre in each assay and the numbers in blue are the number of samples in each quadrant. Samples size can be derived from adding the numbers together.

Supplementary Figure 2. Flow chart showing the cohorts included in the study.

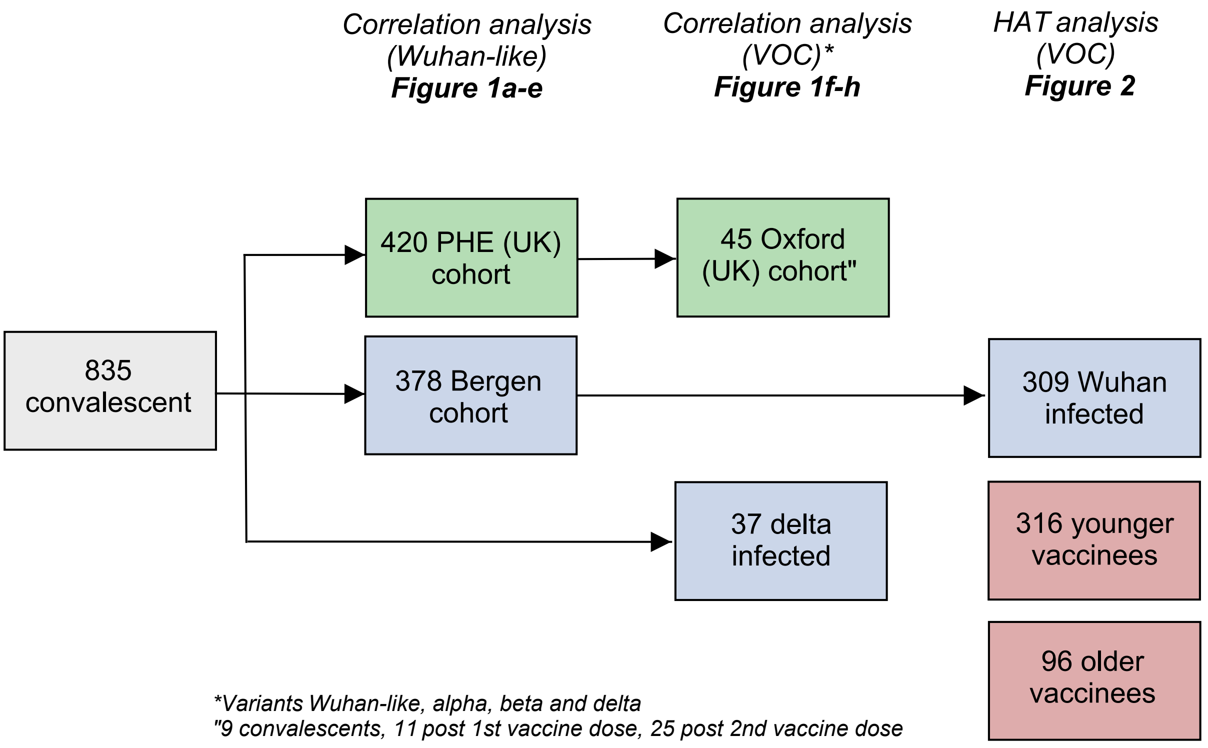

A schematic figure showing the number of individuals used in the Bergen, Public Health England (PHE) and Oxford cohorts used in this study in different analysis and figures.
