# Supplementary material for: A rapid antibody screening haemagglutination test for predicting immunity to SARS-CoV-2 variants of concern
Source: Commun Med (Lond). 2022 Apr 5;2:36. doi: 10.1038/s43856-022-00091-x (PMC9053181; doi:10.1038/s43856-022-00091-x)
Supplement: Supplementary file 4 — Description of Additional Supplementary Files [file 43856_2022_91_MOESM4_ESM.pdf]

Description of Additional Supplementary Files

File Name: Supplementary Data

Description: Raw data behind figures
